# Editorial: Social and physical ecologies for child resilience: wisdom from Asia and Africa

**DOI:** 10.3389/fpsyg.2023.1312299

**Published:** 2023-10-26

**Authors:** Haibin Li, Guanglun Michael Mu, Linda Theron

**Affiliations:** ^1^Institute of Public Policy, South China University of Technology, Guangzhou, China; ^2^Centre for Research in Educational and Social Inclusion, Education Futures, University of South Australia, Adelaide, SA, Australia; ^3^Department of Educational Psychology, Center for the Study of Resilience, University of Pretoria, Pretoria, South Africa

**Keywords:** child and youth resilience, multisystemic resilience, physical ecologies, social ecologies, Asia, Africa

## 1. Introduction

The past five decades have seen significant advancements in child and youth resilience research. However, there can be no room for complacency as the wellbeing of the next generation is continuously thwarted by “large-scale volatilities”, including global economic shocks, geopolitical tensions, the climate emergency, and persistent social inequalities. Hence, we urgently need a proactive approach to build resilience in future generations. It is this urgency that prompted this Research Topic, which explores the “ecologies” that nurture child and youth resilience in Africa and Asia. While Africa and Asia constitute the bulk of human society and are particularly vulnerable to volatilities, they are under-represented in the resilience literature (Theron and van Breda, 2021). This Research Topic is, therefore, crucial to redress the problem of a “marginalized majority” in the production of knowledge about child and youth resilience. In this Research Topic, the term “child and youth” is used to denote the age group of 3–26. It is by no means our intention to lump together young people as a monolithic whole. The use of “child and youth” here cannot be treated as a homogenizing concept but as a pragmatic terminology to cover the age range of the research samples of the different studies included in this Research Topic.

Resilience among young people is a contentious construct, defended and debated through psychological, anthropological, (epi)genetic, and sociological lenses (see review in Mu, 2022). Even so, there is a common understanding that young people's capacity to respond adaptively to significant stress is *co-informed* by their social ecologies, including families, schools, communities, and governments (Mu, 2018), and their built and natural environments (Ungar and Theron, 2020). In other words, systems work most effectively through a multi-systemic, coordinated approach (Ungar and Theron, 2020; Masten et al., 2021). In line with these understandings, the nine articles that constitute this Research Topic take a social-ecological approach to child and youth resilience.

## 2. Overview of the Research Topic

The first two articles position the global COVID-19 pandemic as a critical learning moment for building child resilience in China (Dou et al.) and Singapore (Chen and Yeung). Although the two studies concerned different age groups of children (grades 4–7 schoolers in China and young children aged 3–6 in Singapore) and considered resilience through different sets of variables, they both provided longitudinal, large-scale evidence that highlights the significant role of family functioning/familiar resources in shaping children's resilience in Asian contexts.

The remaining seven articles zoom in on diverse African contexts, providing insights into the importance of multiple, contextually responsive, ecological resources in fostering resilience to diverse stress exposures (e.g., streetism, HIV-related adversities, divorced families, and structural disadvantage). Pillay's review suggests the value of psychological, social, and physical ecologies on child resilience globally, while resilience building at the individual, relationship, community, and societal levels benefited children in South Africa. Similarly, Malindi and Hay affirmed personal strengths and socioecological resources as a booster of resilience for their sampled participants (aged 12–19) brought up in out-of-home care institutions during COVID-challenged times in South Africa. In contrast, Somefun et al. investigated how well benevolent childhood experiences (BCEs) associated with family, school, and community ecologies facilitated resilience to depressive symptoms among young South Africans (aged 18–26) and found no significant association between BCEs and depressive symptoms of the young adult participants. The authors theorized that the measurement of more culturally sensitive BCEs might have produced different results.

Articles in other African contexts indicate protective individual and ecological effects on child and youth resilience. The cross-national study of participants aged between 12 and 20 years in nine sub-Saharan countries (Bandeira et al.) revealed that feeling safe (at home, in the community, and/or at school) was a common enabler of resilience. Goodman et al.'s intervention study on Kenyan children (average age 13) living in street situations found that reintegrating these children into the broader community had greater success when families and communities were supported to provide better care for the children. Similarly, ecological support from the extended family members, peer groups, schools, and the wider communities co-nurtured resilience in children (aged 9–12) exposed to parental divorce in Namibia (Van Schalkwyk and Gentz). Likewise, in the photovoice study reported by Vindevogel and Kimera, the wellbeing of Ugandan young people (aged 14–21) living with HIV was rooted in multisystemic resilience resources in their social and physical ecologies.

## 3. Conclusions

This Research Topic has advanced insights into the multiple ecologies informing child and youth resilience in Asian and African countries. It highlighted the importance of policymakers and practitioners taking a culturally responsive, ecological approach to building and sustaining child and youth resilience (Ungar and Theron, 2020). While efforts to promote child and youth resilience should be tailored to the unique context/s of sub-populations of young people, we anticipate that this Research Topic can have implications beyond the Asian and African settings. However, across the nine articles, very little attention was paid to physical ecologies. Going forward, and as presaged by Ungar and Theron (2020) and Masten et al. (2021), we need research in Africa and Asia that investigates the social and physical ecologies that matter for the resilience of African and Asian young people and the ways in which these ecologies co-facilitate positive outcomes.

## Author contributions

HL: Writing—original draft, Writing—review & editing. GM: Conceptualization, Writing—review & editing. LT: Conceptualization, Writing—review & editing.
